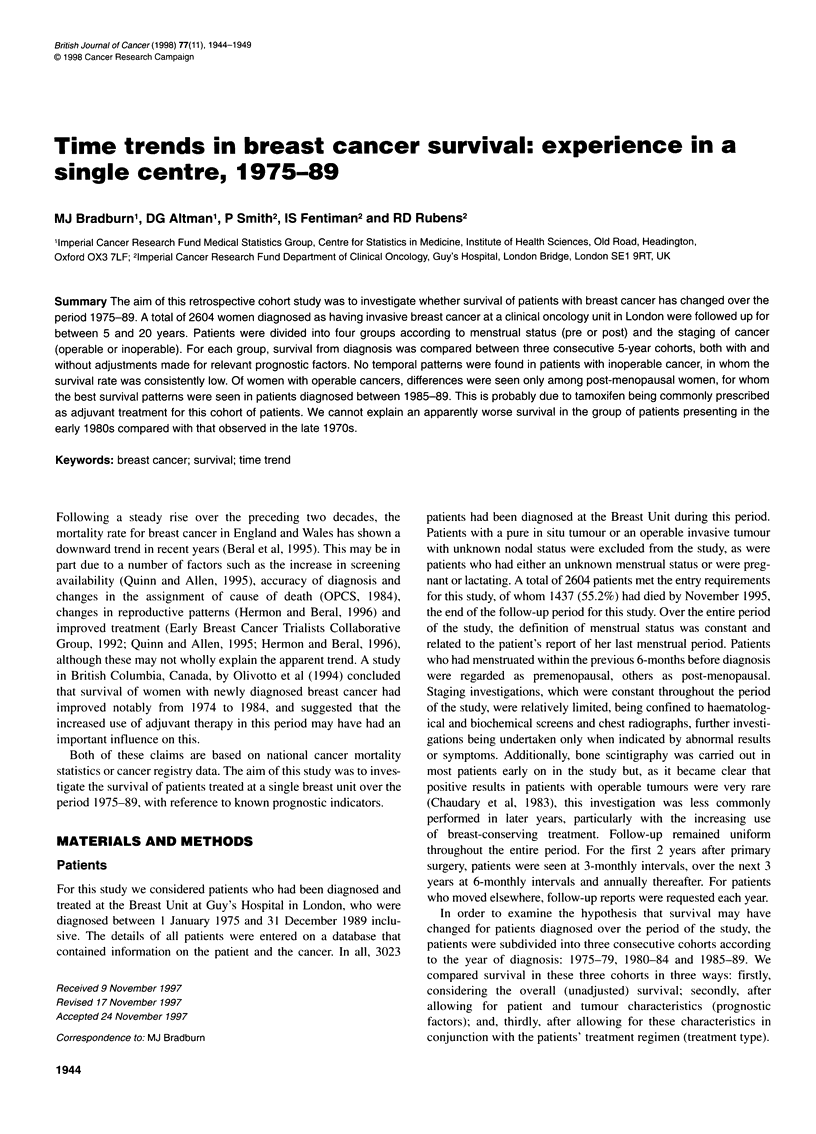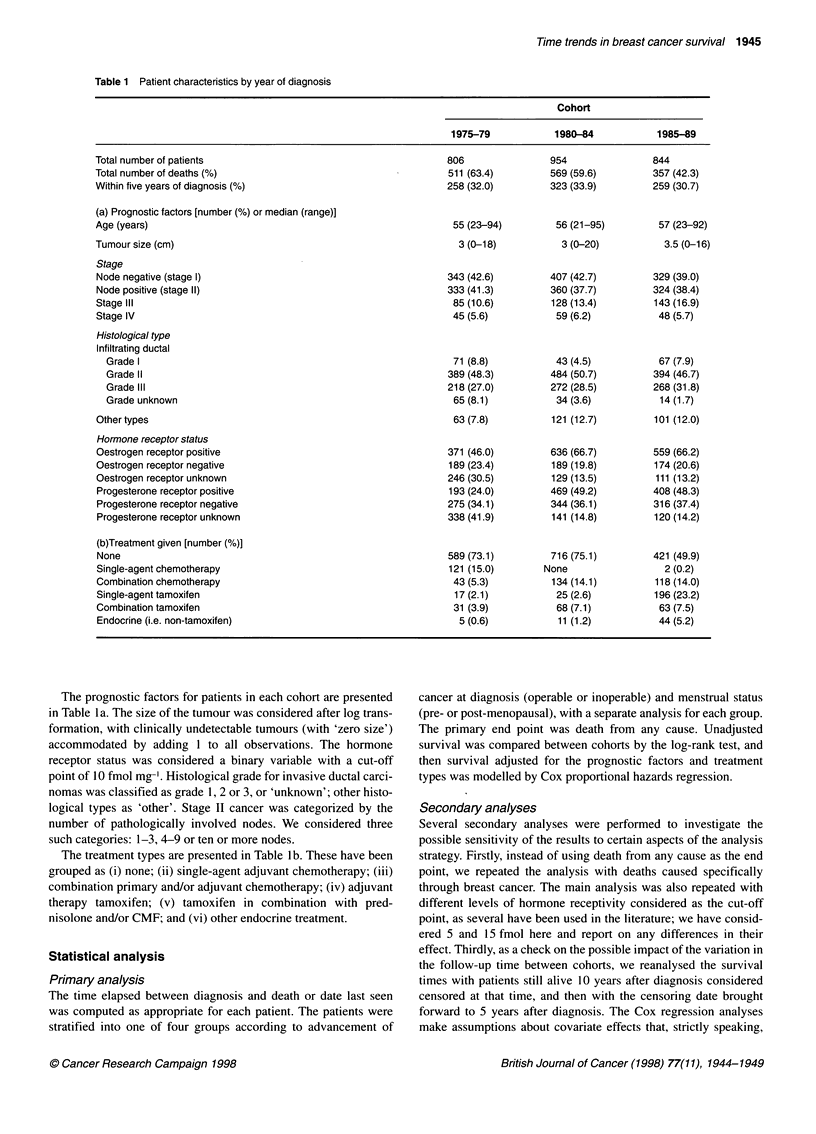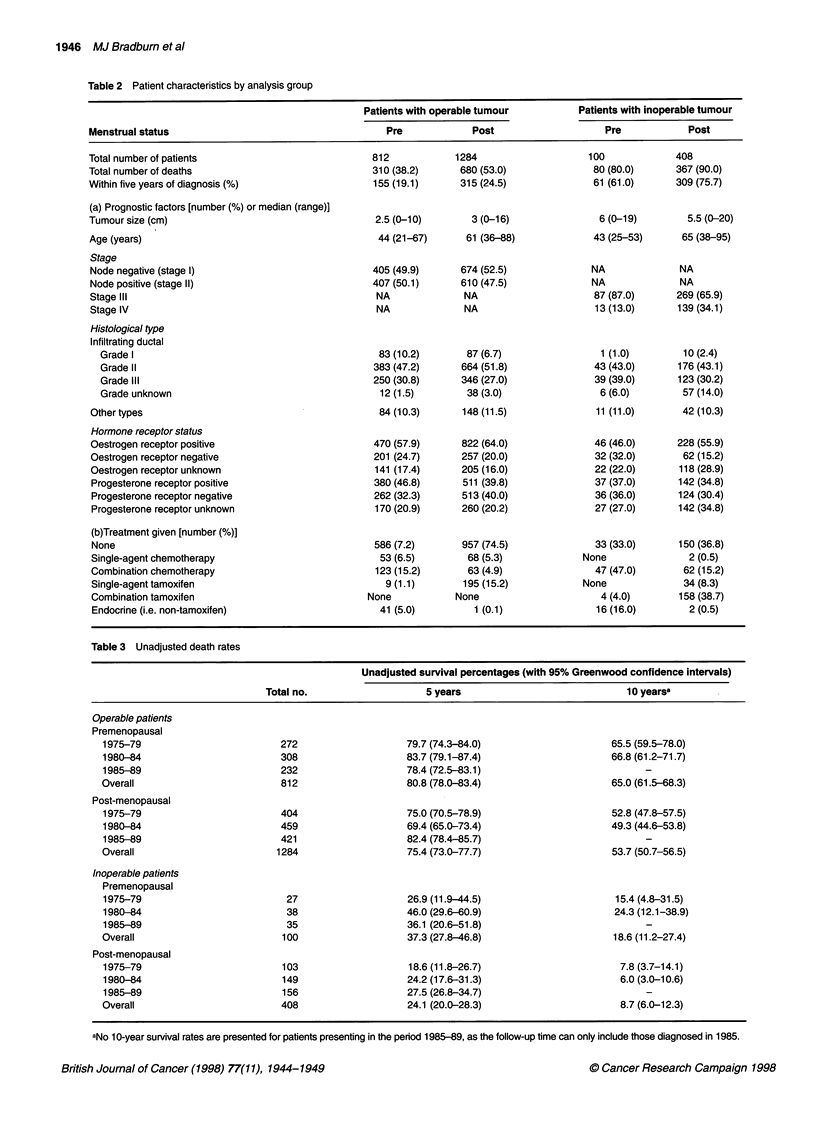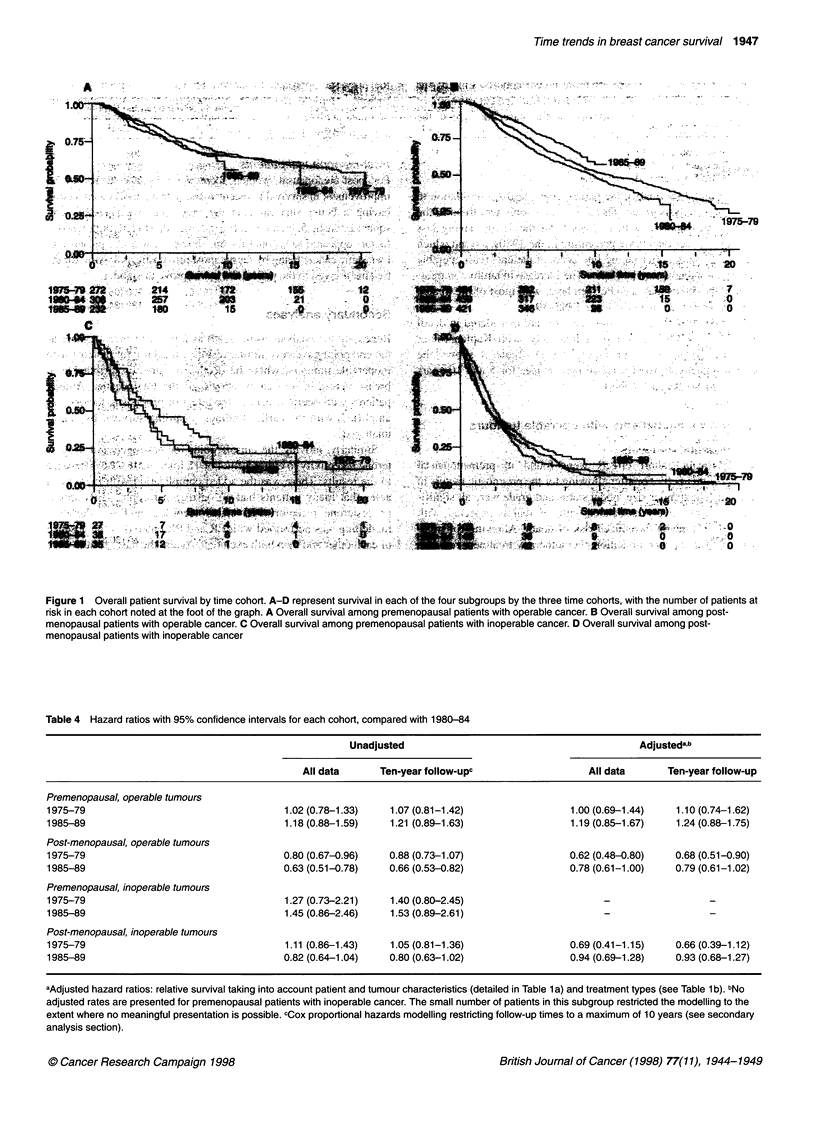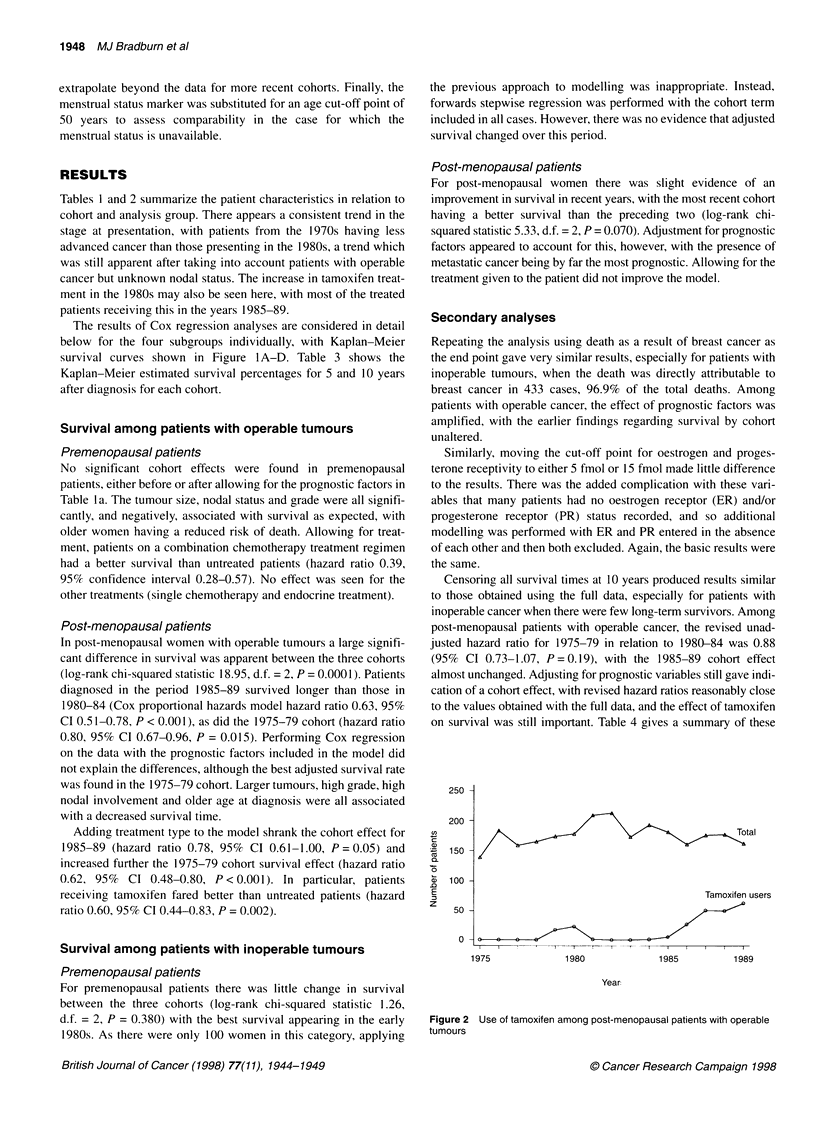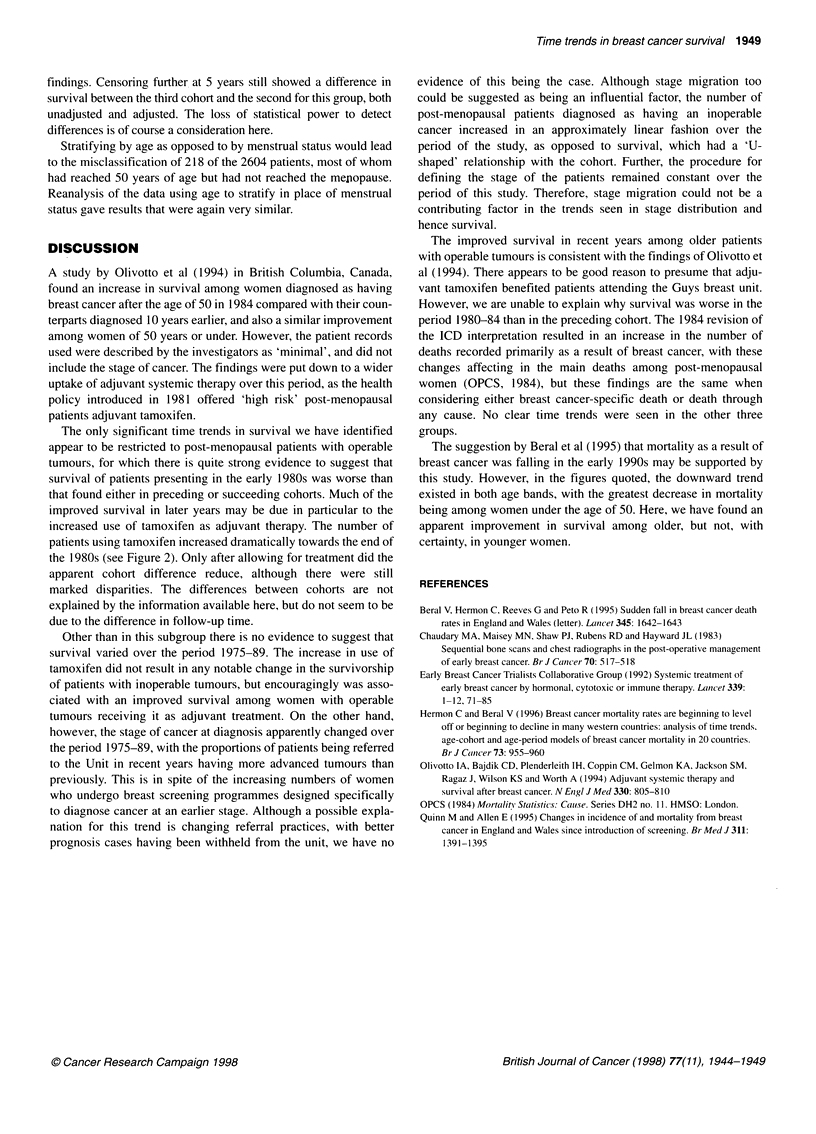# Time trends in breast cancer survival: experience in a single centre, 1975-89.

**DOI:** 10.1038/bjc.1998.322

**Published:** 1998-06

**Authors:** M. J. Bradburn, D. G. Altman, P. Smith, I. S. Fentiman, R. D. Rubens

**Affiliations:** Imperial Cancer Research Fund Medical Statistics Group, Centre for Statistics in Medicine, Institute of Health Sciences, Oxford, UK.

## Abstract

The aim of this retrospective cohort study was to investigate whether survival of patients with breast cancer has changed over the period 1975-89. A total of 2604 women diagnosed as having invasive breast cancer at a clinical oncology unit in London were followed up for between 5 and 20 years. Patients were divided into four groups according to menstrual status (pre or post) and the staging of cancer (operable or inoperable). For each group, survival from diagnosis was compared between three consecutive 5-year cohorts, both with and without adjustments made for relevant prognostic factors. No temporal patterns were found in patients with inoperable cancer, in whom the survival rate was consistently low. Of women with operable cancers, differences were seen only among post-menopausal women, for whom the best survival patterns were seen in patients diagnosed between 1985-89. This is probably due to tamoxifen being commonly prescribed as adjuvant treatment for this cohort of patients. We cannot explain an apparently worse survival in the group of patients presenting in the early 1980s compared with that observed in the late 1970s.


British Journal of Cancer (1998) 77(11), 1944–1949
© 1998 Cancer Research Campaign

### Time trends in breast cancer survival: experience in a single centre, 1975–89

**MJ Bradburn[1], DG Altman[1], P Smith[2], IS Fentiman[2] and RD Rubens[2]**

[1]Imperial Cancer Research Fund Medical Statistics Group, Centre for Statistics in Medicine, Institute of Health Sciences, Old Road, Headington, Oxford OX3 7LF; [2]Imperial Cancer Research Fund Department of Clinical Oncology, Guy's Hospital, London Bridge, London SE1 9RT, UK

**Summary** The aim of this retrospective cohort study was to investigate whether survival of patients with breast cancer has changed over the period 1975–89. A total of 2604 women diagnosed as having invasive breast cancer at a clinical oncology unit in London were followed up for between 5 and 20 years. Patients were divided into four groups according to menstrual status (pre or post) and the staging of cancer (operable or inoperable). For each group, survival from diagnosis was compared between three consecutive 5-year cohorts, both with and without adjustments made for relevant prognostic factors. No temporal patterns were found in patients with inoperable cancer, in whom the survival rate was consistently low. Of women with operable cancers, differences were seen only among post-menopausal women, for whom the best survival patterns were seen in patients diagnosed between 1985–89. This is probably due to tamoxifen being commonly prescribed as adjuvant treatment for this cohort of patients. We cannot explain an apparently worse survival in the group of patients presenting in the early 1980s compared with that observed in the late 1970s.

**Keywords:** breast cancer; survival; time trend

Following a steady rise over the preceding two decades, the mortality rate for breast cancer in England and Wales has shown a downward trend in recent years (Beral et al, 1995). This may be in part due to a number of factors such as the increase in screening availability (Quinn and Allen, 1995), accuracy of diagnosis and changes in the assignment of cause of death (OPCS, 1984), changes in reproductive patterns (Hermon and Beral, 1996) and improved treatment (Early Breast Cancer Trialists Collaborative Group, 1992; Quinn and Allen, 1995; Hermon and Beral, 1996), although these may not wholly explain the apparent trend. A study in British Columbia, Canada, by Olivotto et al (1994) concluded that survival of women with newly diagnosed breast cancer had improved notably from 1974 to 1984, and suggested that the increased use of adjuvant therapy in this period may have had an important influence on this.

Both of these claims are based on national cancer mortality statistics or cancer registry data. The aim of this study was to investigate the survival of patients treated at a single breast unit over the period 1975–89, with reference to known prognostic indicators.

### MATERIALS AND METHODS

### Patients

For this study we considered patients who had been diagnosed and treated at the Breast Unit at Guy's Hospital in London, who were diagnosed between 1 January 1975 and 31 December 1989 inclusive. The details of all patients were entered on a database that contained information on the patient and the cancer. In all, 3023 patients had been diagnosed at the Breast Unit during this period. Patients with a pure in situ tumour or an operable invasive tumour with unknown nodal status were excluded from the study, as were patients who had either an unknown menstrual status or were pregnant or lactating. A total of 2604 patients met the entry requirements for this study, of whom 1437 (55.2%) had died by November 1995, the end of the follow-up period for this study. Over the entire period of the study, the definition of menstrual status was constant and related to the patient's report of her last menstrual period. Patients who had menstruated within the previous 6-months before diagnosis were regarded as premenopausal, others as post-menopausal. Staging investigations, which were constant throughout the period of the study, were relatively limited, being confined to haematological and biochemical screens and chest radiographs, further investigations being undertaken only when indicated by abnormal results or symptoms. Additionally, bone scintigraphy was carried out in most patients early on in the study but, as it became clear that positive results in patients with operable tumours were very rare (Chaudary et al, 1983), this investigation was less commonly performed in later years, particularly with the increasing use of breast-conserving treatment. Follow-up remained uniform throughout the entire period. For the first 2 years after primary surgery, patients were seen at 3-monthly intervals, over the next 3 years at 6-monthly intervals and annually thereafter. For patients who moved elsewhere, follow-up reports were requested each year.

In order to examine the hypothesis that survival may have changed for patients diagnosed over the period of the study, the patients were subdivided into three consecutive cohorts according to the year of diagnosis: 1975–79, 1980–84 and 1985–89. We compared survival in these three cohorts in three ways: firstly, considering the overall (unadjusted) survival; secondly, after allowing for patient and tumour characteristics (prognostic factors); and, thirdly, after allowing for these characteristics in conjunction with the patients' treatment regimen (treatment type).

*Received 9 November 1997*
*Revised 17 November 1997*
*Accepted 24 November 1997*

*Correspondence to:* MJ Bradburn

**Table 1** Patient characteristics by year of diagnosis

| | Cohort | | |
|---|---|---|---|
| | **1975–79** | **1980–84** | **1985–89** |
| Total number of patients | 806 | 954 | 844 |
| Total number of deaths (%) | 511 (63.4) | 569 (59.6) | 357 (42.3) |
| Within five years of diagnosis (%) | 258 (32.0) | 323 (33.9) | 259 (30.7) |
| (a) Prognostic factors [number (%) or median (range)] | | | |
| Age (years) | 55 (23–94) | 56 (21–95) | 57 (23–92) |
| Tumour size (cm) | 3 (0–18) | 3 (0–20) | 3.5 (0–16) |
| *Stage* | | | |
| Node negative (stage I) | 343 (42.6) | 407 (42.7) | 329 (39.0) |
| Node positive (stage II) | 333 (41.3) | 360 (37.7) | 324 (38.4) |
| Stage III | 85 (10.6) | 128 (13.4) | 143 (16.9) |
| Stage IV | 45 (5.6) | 59 (6.2) | 48 (5.7) |
| *Histological type* | | | |
| Infiltrating ductal | | | |
| Grade I | 71 (8.8) | 43 (4.5) | 67 (7.9) |
| Grade II | 389 (48.3) | 484 (50.7) | 394 (46.7) |
| Grade III | 218 (27.0) | 272 (28.5) | 268 (31.8) |
| Grade unknown | 65 (8.1) | 34 (3.6) | 14 (1.7) |
| Other types | 63 (7.8) | 121 (12.7) | 101 (12.0) |
| *Hormone receptor status* | | | |
| Oestrogen receptor positive | 371 (46.0) | 636 (66.7) | 559 (66.2) |
| Oestrogen receptor negative | 189 (23.4) | 189 (19.8) | 174 (20.6) |
| Oestrogen receptor unknown | 246 (30.5) | 129 (13.5) | 111 (13.2) |
| Progesterone receptor positive | 193 (24.0) | 469 (49.2) | 408 (48.3) |
| Progesterone receptor negative | 275 (34.1) | 344 (36.1) | 316 (37.4) |
| Progesterone receptor unknown | 338 (41.9) | 141 (14.8) | 120 (14.2) |
| (b)Treatment given [number (%)] | | | |
| None | 589 (73.1) | 716 (75.1) | 421 (49.9) |
| Single-agent chemotherapy | 121 (15.0) | None | 2 (0.2) |
| Combination chemotherapy | 43 (5.3) | 134 (14.1) | 118 (14.0) |
| Single-agent tamoxifen | 17 (2.1) | 25 (2.6) | 196 (23.2) |
| Combination tamoxifen | 31 (3.9) | 68 (7.1) | 63 (7.5) |
| Endocrine (i.e. non-tamoxifen) | 5 (0.6) | 11 (1.2) | 44 (5.2) |

The prognostic factors for patients in each cohort are presented in Table 1a. The size of the tumour was considered after log transformation, with clinically undetectable tumours (with 'zero size') accommodated by adding 1 to all observations. The hormone receptor status was considered a binary variable with a cut-off point of 10 fmol $mg^{-1}$. Histological grade for invasive ductal carcinomas was classified as grade 1, 2 or 3, or 'unknown'; other histological types as 'other'. Stage II cancer was categorized by the number of pathologically involved nodes. We considered three such categories: 1–3, 4–9 or ten or more nodes.

The treatment types are presented in Table 1b. These have been grouped as (i) none; (ii) single-agent adjuvant chemotherapy; (iii) combination primary and/or adjuvant chemotherapy; (iv) adjuvant therapy tamoxifen; (v) tamoxifen in combination with prednisolone and/or CMF; and (vi) other endocrine treatment.

### Statistical analysis

### *Primary analysis*

The time elapsed between diagnosis and death or date last seen was computed as appropriate for each patient. The patients were stratified into one of four groups according to advancement of cancer at diagnosis (operable or inoperable) and menstrual status (pre- or post-menopausal), with a separate analysis for each group. The primary end point was death from any cause. Unadjusted survival was compared between cohorts by the log-rank test, and then survival adjusted for the prognostic factors and treatment types was modelled by Cox proportional hazards regression.

### *Secondary analyses*

Several secondary analyses were performed to investigate the possible sensitivity of the results to certain aspects of the analysis strategy. Firstly, instead of using death from any cause as the end point, we repeated the analysis with deaths caused specifically through breast cancer. The main analysis was also repeated with different levels of hormone receptivity considered as the cut-off point, as several have been used in the literature; we have considered 5 and 15 fmol here and report on any differences in their effect. Thirdly, as a check on the possible impact of the variation in the follow-up time between cohorts, we reanalysed the survival times with patients still alive 10 years after diagnosis considered censored at that time, and then with the censoring date brought forward to 5 years after diagnosis. The Cox regression analyses make assumptions about covariate effects that, strictly speaking,

© Cancer Research Campaign 1998 

**Table 2** Patient characteristics by analysis group

| Menstrual status | Patients with operable tumour Pre | Patients with operable tumour Post | Patients with inoperable tumour Pre | Patients with inoperable tumour Post |
|---|---|---|---|---|
| Total number of patients | 812 | 1284 | 100 | 408 |
| Total number of deaths | 310 (38.2) | 680 (53.0) | 80 (80.0) | 367 (90.0) |
| Within five years of diagnosis (%) | 155 (19.1) | 315 (24.5) | 61 (61.0) | 309 (75.7) |
| (a) Prognostic factors [number (%) or median (range)] | | | | |
| Tumour size (cm) | 2.5 (0–10) | 3 (0–16) | 6 (0–19) | 5.5 (0–20) |
| Age (years) | 44 (21–67) | 61 (36–88) | 43 (25–53) | 65 (38–95) |
| *Stage* | | | | |
| Node negative (stage I) | 405 (49.9) | 674 (52.5) | NA | NA |
| Node positive (stage II) | 407 (50.1) | 610 (47.5) | NA | NA |
| Stage III | NA | NA | 87 (87.0) | 269 (65.9) |
| Stage IV | NA | NA | 13 (13.0) | 139 (34.1) |
| *Histological type* | | | | |
| Infiltrating ductal | | | | |
| Grade I | 83 (10.2) | 87 (6.7) | 1 (1.0) | 10 (2.4) |
| Grade II | 383 (47.2) | 664 (51.8) | 43 (43.0) | 176 (43.1) |
| Grade III | 250 (30.8) | 346 (27.0) | 39 (39.0) | 123 (30.2) |
| Grade unknown | 12 (1.5) | 38 (3.0) | 6 (6.0) | 57 (14.0) |
| Other types | 84 (10.3) | 148 (11.5) | 11 (11.0) | 42 (10.3) |
| *Hormone receptor status* | | | | |
| Oestrogen receptor positive | 470 (57.9) | 822 (64.0) | 46 (46.0) | 228 (55.9) |
| Oestrogen receptor negative | 201 (24.7) | 257 (20.0) | 32 (32.0) | 62 (15.2) |
| Oestrogen receptor unknown | 141 (17.4) | 205 (16.0) | 22 (22.0) | 118 (28.9) |
| Progesterone receptor positive | 380 (46.8) | 511 (39.8) | 37 (37.0) | 142 (34.8) |
| Progesterone receptor negative | 262 (32.3) | 513 (40.0) | 36 (36.0) | 124 (30.4) |
| Progesterone receptor unknown | 170 (20.9) | 260 (20.2) | 27 (27.0) | 142 (34.8) |
| (b)Treatment given [number (%)] | | | | |
| None | 586 (7.2) | 957 (74.5) | 33 (33.0) | 150 (36.8) |
| Single-agent chemotherapy | 53 (6.5) | 68 (5.3) | None | 2 (0.5) |
| Combination chemotherapy | 123 (15.2) | 63 (4.9) | 47 (47.0) | 62 (15.2) |
| Single-agent tamoxifen | 9 (1.1) | 195 (15.2) | None | 34 (8.3) |
| Combination tamoxifen | None | None | 4 (4.0) | 158 (38.7) |
| Endocrine (i.e. non-tamoxifen) | 41 (5.0) | 1 (0.1) | 16 (16.0) | 2 (0.5) |

**Table 3** Unadjusted death rates

| | Total no. | Unadjusted survival percentages (with 95% Greenwood confidence intervals) 5 years | 10 years[a] |
|---|---|---|---|
| *Operable patients* | | | |
| Premenopausal | | | |
| 1975–79 | 272 | 79.7 (74.3–84.0) | 65.5 (59.5–78.0) |
| 1980–84 | 308 | 83.7 (79.1–87.4) | 66.8 (61.2–71.7) |
| 1985–89 | 232 | 78.4 (72.5–83.1) | – |
| Overall | 812 | 80.8 (78.0–83.4) | 65.0 (61.5–68.3) |
| Post-menopausal | | | |
| 1975–79 | 404 | 75.0 (70.5–78.9) | 52.8 (47.8–57.5) |
| 1980–84 | 459 | 69.4 (65.0–73.4) | 49.3 (44.6–53.8) |
| 1985–89 | 421 | 82.4 (78.4–85.7) | – |
| Overall | 1284 | 75.4 (73.0–77.7) | 53.7 (50.7–56.5) |
| *Inoperable patients* | | | |
| Premenopausal | | | |
| 1975–79 | 27 | 26.9 (11.9–44.5) | 15.4 (4.8–31.5) |
| 1980–84 | 38 | 46.0 (29.6–60.9) | 24.3 (12.1–38.9) |
| 1985–89 | 35 | 36.1 (20.6–51.8) | – |
| Overall | 100 | 37.3 (27.8–46.8) | 18.6 (11.2–27.4) |
| Post-menopausal | | | |
| 1975–79 | 103 | 18.6 (11.8–26.7) | 7.8 (3.7–14.1) |
| 1980–84 | 149 | 24.2 (17.6–31.3) | 6.0 (3.0–10.6) |
| 1985–89 | 156 | 27.5 (26.8–34.7) | – |
| Overall | 408 | 24.1 (20.0–28.3) | 8.7 (6.0–12.3) |

[a]No 10-year survival rates are presented for patients presenting in the period 1985–89, as the follow-up time can only include those diagnosed in 1985.

 *© Cancer Research Campaign 1998*

**Figure 1** Overall patient survival by time cohort. **A–D** represent survival in each of the four subgroups by the three time cohorts, with the number of patients at risk in each cohort noted at the foot of the graph. **A** Overall survival among premenopausal patients with operable cancer. **B** Overall survival among post-menopausal patients with operable cancer. **C** Overall survival among premenopausal patients with inoperable cancer. **D** Overall survival among post-menopausal patients with inoperable cancer

**Table 4** Hazard ratios with 95% confidence intervals for each cohort, compared with 1980–84

| | **Unadjusted** | | **Adjusted[a,b]** | |
|---|---|---|---|---|
| | **All data** | **Ten-year follow-up[c]** | **All data** | **Ten-year follow-up** |
| *Premenopausal, operable tumours* | | | | |
| 1975–79 | 1.02 (0.78–1.33) | 1.07 (0.81–1.42) | 1.00 (0.69–1.44) | 1.10 (0.74–1.62) |
| 1985–89 | 1.18 (0.88–1.59) | 1.21 (0.89–1.63) | 1.19 (0.85–1.67) | 1.24 (0.88–1.75) |
| *Post-menopausal, operable tumours* | | | | |
| 1975–79 | 0.80 (0.67–0.96) | 0.88 (0.73–1.07) | 0.62 (0.48–0.80) | 0.68 (0.51–0.90) |
| 1985–89 | 0.63 (0.51–0.78) | 0.66 (0.53–0.82) | 0.78 (0.61–1.00) | 0.79 (0.61–1.02) |
| *Premenopausal, inoperable tumours* | | | | |
| 1975–79 | 1.27 (0.73–2.21) | 1.40 (0.80–2.45) | – | – |
| 1985–89 | 1.45 (0.86–2.46) | 1.53 (0.89–2.61) | – | – |
| *Post-menopausal, inoperable tumours* | | | | |
| 1975–79 | 1.11 (0.86–1.43) | 1.05 (0.81–1.36) | 0.69 (0.41–1.15) | 0.66 (0.39–1.12) |
| 1985–89 | 0.82 (0.64–1.04) | 0.80 (0.63–1.02) | 0.94 (0.69–1.28) | 0.93 (0.68–1.27) |

[a]Adjusted hazard ratios: relative survival taking into account patient and tumour characteristics (detailed in Table 1a) and treatment types (see Table 1b). [b]No adjusted rates are presented for premenopausal patients with inoperable cancer. The small number of patients in this subgroup restricted the modelling to the extent where no meaningful presentation is possible. [c]Cox proportional hazards modelling restricting follow-up times to a maximum of 10 years (see secondary analysis section).

© Cancer Research Campaign 1998

extrapolate beyond the data for more recent cohorts. Finally, the menstrual status marker was substituted for an age cut-off point of 50 years to assess comparability in the case for which the menstrual status is unavailable.

### RESULTS

Tables 1 and 2 summarize the patient characteristics in relation to cohort and analysis group. There appears a consistent trend in the stage at presentation, with patients from the 1970s having less advanced cancer than those presenting in the 1980s, a trend which was still apparent after taking into account patients with operable cancer but unknown nodal status. The increase in tamoxifen treatment in the 1980s may also be seen here, with most of the treated patients receiving this in the years 1985–89.

The results of Cox regression analyses are considered in detail below for the four subgroups individually, with Kaplan–Meier survival curves shown in Figure 1A–D. Table 3 shows the Kaplan–Meier estimated survival percentages for 5 and 10 years after diagnosis for each cohort.

### Survival among patients with operable tumours

*Premenopausal patients*

No significant cohort effects were found in premenopausal patients, either before or after allowing for the prognostic factors in Table 1a. The tumour size, nodal status and grade were all significantly, and negatively, associated with survival as expected, with older women having a reduced risk of death. Allowing for treatment, patients on a combination chemotherapy treatment regimen had a better survival than untreated patients (hazard ratio 0.39, 95% confidence interval 0.28–0.57). No effect was seen for the other treatments (single chemotherapy and endocrine treatment).

*Post-menopausal patients*

In post-menopausal women with operable tumours a large significant difference in survival was apparent between the three cohorts (log-rank chi-squared statistic 18.95, d.f. = 2, $P = 0.0001$). Patients diagnosed in the period 1985–89 survived longer than those in 1980–84 (Cox proportional hazards model hazard ratio 0.63, 95% CI 0.51–0.78, $P < 0.001$), as did the 1975–79 cohort (hazard ratio 0.80, 95% CI 0.67–0.96, $P = 0.015$). Performing Cox regression on the data with the prognostic factors included in the model did not explain the differences, although the best adjusted survival rate was found in the 1975–79 cohort. Larger tumours, high grade, high nodal involvement and older age at diagnosis were all associated with a decreased survival time.

Adding treatment type to the model shrank the cohort effect for 1985–89 (hazard ratio 0.78, 95% CI 0.61–1.00, $P = 0.05$) and increased further the 1975–79 cohort survival effect (hazard ratio 0.62, 95% CI 0.48–0.80, $P < 0.001$). In particular, patients receiving tamoxifen fared better than untreated patients (hazard ratio 0.60, 95% CI 0.44–0.83, $P = 0.002$).

### Survival among patients with inoperable tumours

*Premenopausal patients*

For premenopausal patients there was little change in survival between the three cohorts (log-rank chi-squared statistic 1.26, d.f. = 2, $P = 0.380$) with the best survival appearing in the early 1980s. As there were only 100 women in this category, applying the previous approach to modelling was inappropriate. Instead, forwards stepwise regression was performed with the cohort term included in all cases. However, there was no evidence that adjusted survival changed over this period.

*Post-menopausal patients*

For post-menopausal women there was slight evidence of an improvement in survival in recent years, with the most recent cohort having a better survival than the preceding two (log-rank chi-squared statistic 5.33, d.f. = 2, $P = 0.070$). Adjustment for prognostic factors appeared to account for this, however, with the presence of metastatic cancer being by far the most prognostic. Allowing for the treatment given to the patient did not improve the model.

### Secondary analyses

Repeating the analysis using death as a result of breast cancer as the end point gave very similar results, especially for patients with inoperable tumours, when the death was directly attributable to breast cancer in 433 cases, 96.9% of the total deaths. Among patients with operable cancer, the effect of prognostic factors was amplified, with the earlier findings regarding survival by cohort unaltered.

Similarly, moving the cut-off point for oestrogen and progesterone receptivity to either 5 fmol or 15 fmol made little difference to the results. There was the added complication with these variables that many patients had no oestrogen receptor (ER) and/or progesterone receptor (PR) status recorded, and so additional modelling was performed with ER and PR entered in the absence of each other and then both excluded. Again, the basic results were the same.

Censoring all survival times at 10 years produced results similar to those obtained using the full data, especially for patients with inoperable cancer when there were few long-term survivors. Among post-menopausal patients with operable cancer, the revised unadjusted hazard ratio for 1975–79 in relation to 1980–84 was 0.88 (95% CI 0.73–1.07, $P = 0.19$), with the 1985–89 cohort effect almost unchanged. Adjusting for prognostic variables still gave indication of a cohort effect, with revised hazard ratios reasonably close to the values obtained with the full data, and the effect of tamoxifen on survival was still important. Table 4 gives a summary of these

**Figure 2** Use of tamoxifen among post-menopausal patients with operable tumours

*© Cancer Research Campaign 1998*

findings. Censoring further at 5 years still showed a difference in survival between the third cohort and the second for this group, both unadjusted and adjusted. The loss of statistical power to detect differences is of course a consideration here.

Stratifying by age as opposed to by menstrual status would lead to the misclassification of 218 of the 2604 patients, most of whom had reached 50 years of age but had not reached the menopause. Reanalysis of the data using age to stratify in place of menstrual status gave results that were again very similar.

### DISCUSSION

A study by Olivotto et al (1994) in British Columbia, Canada, found an increase in survival among women diagnosed as having breast cancer after the age of 50 in 1984 compared with their counterparts diagnosed 10 years earlier, and also a similar improvement among women of 50 years or under. However, the patient records used were described by the investigators as 'minimal', and did not include the stage of cancer. The findings were put down to a wider uptake of adjuvant systemic therapy over this period, as the health policy introduced in 1981 offered 'high risk' post-menopausal patients adjuvant tamoxifen.

The only significant time trends in survival we have identified appear to be restricted to post-menopausal patients with operable tumours, for which there is quite strong evidence to suggest that survival of patients presenting in the early 1980s was worse than that found either in preceding or succeeding cohorts. Much of the improved survival in later years may be due in particular to the increased use of tamoxifen as adjuvant therapy. The number of patients using tamoxifen increased dramatically towards the end of the 1980s (see Figure 2). Only after allowing for treatment did the apparent cohort difference reduce, although there were still marked disparities. The differences between cohorts are not explained by the information available here, but do not seem to be due to the difference in follow-up time.

Other than in this subgroup there is no evidence to suggest that survival varied over the period 1975–89. The increase in use of tamoxifen did not result in any notable change in the survivorship of patients with inoperable tumours, but encouragingly was associated with an improved survival among women with operable tumours receiving it as adjuvant treatment. On the other hand, however, the stage of cancer at diagnosis apparently changed over the period 1975–89, with the proportions of patients being referred to the Unit in recent years having more advanced tumours than previously. This is in spite of the increasing numbers of women who undergo breast screening programmes designed specifically to diagnose cancer at an earlier stage. Although a possible explanation for this trend is changing referral practices, with better prognosis cases having been withheld from the unit, we have no evidence of this being the case. Although stage migration too could be suggested as being an influential factor, the number of post-menopausal patients diagnosed as having an inoperable cancer increased in an approximately linear fashion over the period of the study, as opposed to survival, which had a 'U-shaped' relationship with the cohort. Further, the procedure for defining the stage of the patients remained constant over the period of this study. Therefore, stage migration could not be a contributing factor in the trends seen in stage distribution and hence survival.

The improved survival in recent years among older patients with operable tumours is consistent with the findings of Olivotto et al (1994). There appears to be good reason to presume that adjuvant tamoxifen benefited patients attending the Guys breast unit. However, we are unable to explain why survival was worse in the period 1980–84 than in the preceding cohort. The 1984 revision of the ICD interpretation resulted in an increase in the number of deaths recorded primarily as a result of breast cancer, with these changes affecting in the main deaths among post-menopausal women (OPCS, 1984), but these findings are the same when considering either breast cancer-specific death or death through any cause. No clear time trends were seen in the other three groups.

The suggestion by Beral et al (1995) that mortality as a result of breast cancer was falling in the early 1990s may be supported by this study. However, in the figures quoted, the downward trend existed in both age bands, with the greatest decrease in mortality being among women under the age of 50. Here, we have found an apparent improvement in survival among older, but not, with certainty, in younger women.

### REFERENCES

Beral V, Hermon C, Reeves G and Peto R (1995) Sudden fall in breast cancer death rates in England and Wales (letter). *Lancet* **345**: 1642–1643

Chaudary MA, Maisey MN, Shaw PJ, Rubens RD and Hayward JL (1983) Sequential bone scans and chest radiographs in the post-operative management of early breast cancer. *Br J Cancer* **70**: 517–518

Early Breast Cancer Trialists Collaborative Group (1992) Systemic treatment of early breast cancer by hormonal, cytotoxic or immune therapy. *Lancet* **339**: 1–12, 71–85

Hermon C and Beral V (1996) Breast cancer mortality rates are beginning to level off or beginning to decline in many western countries: analysis of time trends, age-cohort and age-period models of breast cancer mortality in 20 countries. *Br J Cancer* **73**: 955–960

Olivotto IA, Bajdik CD, Plenderleith IH, Coppin CM, Gelmon KA, Jackson SM, Ragaz J, Wilson KS and Worth A (1994) Adjuvant systemic therapy and survival after breast cancer. *N Engl J Med* **330**: 805–810

OPCS (1984) *Mortality Statistics: Cause*. Series DH2 no. 11. HMSO: London.

Quinn M and Allen E (1995) Changes in incidence of and mortality from breast cancer in England and Wales since introduction of screening. *Br Med J* **311**: 1391–1395

© Cancer Research Campaign 1998